# Sustainability analysis of bioethanol production from grain and tuber starchy feedstocks

**DOI:** 10.1038/s41598-022-24854-7

**Published:** 2022-12-05

**Authors:** A. Sanni, A. S. Olawale, Y. M. Sani, S. Kheawhom

**Affiliations:** 1 Department of Chemical Engineering, Ahmadu Bello University, Zaria, Nigeria; 2 Department of Chemical Engineering, Chulalongkorn University, Bangkok, Thailand

**Keywords:** Biotechnology, Climate sciences, Engineering

## Abstract

A comparative sustainability study of bioethanol production from selected starchy feedstocks that are abundantly available was carried out in this work. This is to ensure the safe, reliable, and efficient production and consumption of fuel-grade bioethanol. The analysis utilised the established economic minimum bioethanol plant capacity of 158,000 m^3^/annum. The processing flowsheet model utilised was the same for each feedstock. The sustainability study's economic, environmental, and energy perspectives were investigated. The economic and environmental indices were assessed using Superpro Designer and openLCA sustainability software, respectively. Exergy and lost work were subsequently estimated manually with Microsoft Excel. The economic analyses showed that the plants using cassava and sweet potato initially had the highest return on investment (ROI) of 64.41 and 41.96% respectively at a minimum of 80% plants’ capacity utilisation. The break-even point occurs at a bioethanol price of $3.27 per gallon, beyond which positive net present values were obtained for the four processes. The least profitable plant was based on sorghum recording an ROI value of 34.11%. The environmental assessment on the four selected feedstocks showed that the processes based on cassava, corn, sweet potato, and sorghum recorded encouraging global warming potential (GWP) of 0.2452, 0.2067, 2.5261, and 0.2099 kg CO_2_ equivalent respectively. Cassava and corn emerged as the two most economically viable feedstocks when economic parameters were adjusted to include pollutants emission/discharge costs but with a slight decrease in profitability indices. The lost work analyses showed that distillation columns were the least energy-efficient units in the four bioethanol production routes assessed, recording loss work of about 61, 68, 34, and 49 MW for cassava, sweet potato, corn, and sorghum processing plants respectively. However, the net energy balance (NEB) and energy renewability results of the four production routes showed that the processes utilising the four selected starchy biomass feedstocks are more sustainable compared to fossil fuels.

## Introduction

The global energy resources profile is rich in natural gas, solar, tar sand, coal, biomass, and crude oil but fossil fuel-based energy has remained the greatest energy and revenue source^1^. Crude oil accounts for about 53,369 TWh of global power consumption, while natural gas-based global power consumption stands at 39,063 TWh^2^. These figures are products of their constantly increasing consumption levels for the past few decades despite the attractive prospect possessed by other energy sources especially biofuels^2^.

Biofuels have been identified as low-carbon alternatives to fossil fuels because they tend to reduce greenhouse gas (GHG) emissions, and the other associated undesirable climate change impacts^3^. The constantly growing worldwide industrialization and overreliance on non-renewable energy sources have resulted in a huge quantity of greenhouse gases being emitted. This has led to a rise in global temperature and the attendant effect of environmental dilapidation^4^. The increment in worldwide mean atmospheric carbon dioxide (CO_2_) concentration from the pre-industrialization era of 1850 to date has been estimated at 285–419 ppm^5,6^ which is quite significant. In light of this, the United Kingdom meteorological office evaluates a worldwide mean surface temperature rise of about 0.97–1.21 °C between these periods, with a dominant estimate of 1.09 °C. It has also been predicted that this trend of global temperature increase will continue in subsequent years^7^. Furthermore, Rabaey and Ragauskas, 2014 also predicted a rise in global greenhouse gas emissions to be 50% by 2050, as a result of the utilisation of non-renewable energy-based CO_2_ emissions^8^. Therefore, in the absence of urgent and effective measures or technological modifications to curtail CO_2_ emissions, the global mean atmospheric concentration of CO_2_ and ocean temperatures will steadily increase^9,10^.

Fortunately, world leaders are well informed about this environmental deterioration posed by fossil fuel consumption and as such adopted an agendum of cleaner energy of the sustainable development goal in 2015. Some countries like USA and Brazil initiated a plan to realise this agendum by the use of biofuel (gasohol); 10% bioethanol and 90% gasoline (E10) in preference to the use of 100% conventional premium motor spirit (PMS) with much priority given to corn and sugarcane, as feedstocks for the bioethanol production^11^.

The usage of renewable sources for fuels and electricity in transport is capable of profitably achieving great decarbonisation of the transport sector and expanding energy diversification within the sector while encouraging modernization and providing jobs in the Union economy and minimising overreliance on energy importations^12^.

However, the reliance on first-generation (consumables) bioethanol feedstocks has prompted great concern and opinions on whether its fulfillment will not engender the risk of food scarcity^12^. Meanwhile, second-generation biofuel production utilizes uneatable materials but the financial involvement in its large-scale production has hindered its growth. Recently, attention has been shifted to third and fourth-generation biofuels with greater emphasis on the use of algae and genetically modified microorganisms, respectively^12^. These probable interwoven consequences of food shortage from the utilization of food crops to ensure energy security have made the second, third, and fourth-generation feedstocks (lignocellulosic) more attractive^12^. Nevertheless, decisive measures can be put in place by governments and other stakeholders to replace the obsolete traditional farming practices with modern mechanized techniques to ensure improved acreage yields of these food crops for sustainable biofuel policies and food security.

The economic viability of bioethanol production using different feedstocks is dependent on factors such as local cost and composition of the feedstocks, energy cost, technology alternatives, plant capacity, etc. The sustainability of bioethanol production with respect to economic, environmental, and social indices has been carried out for some starchy feedstocks when utilised in different countries and/or under different conditions^3,4,12,13–15^. Consideration of these sustainability indicators requires the stepwise analysis of bioethanol produced from different starchy feedstocks in different climes. Despite this bright global prospect for biofuel production, no work has been reported on the sustainability assessment of bioethanol production from this abundant tuber and grain starchy feedstocks. Thus, sustainability analysis of bioethanol production with these feedstocks is essential to assist policymakers and bioethanol producers in feedstock selection.

In this work, a sustainability study of bioethanol production from cassava, sweet potato, corn, and sorghum was carried out as well as a comparative assessment of the technological pathways utilising the feedstocks.

## Methodology

### Process technology selection

The dry grind milling method was chosen for the starchy feedstocks because it has been identified to be cheaper and produce a higher yield of bioethanol than the wet milling process^16^. Separate hydrolysis and fermentation (SHF) was selected in preference to simultaneous saccharification and fermentation (SSF) for maximum starch-to-glucose conversion^17^. Two enzymes in separate reactors (alpha-amylase in alkaline (ammonia) medium, the temperature of 100 °C, a pressure of 1.013 bar, and 0.082% dried base) and glucoamylase (in sulphuric acid medium, the temperature of 60 °C, pressure of 1.013 bar, and 0.11% dried base) was selected for saccharification^18^. The selected temperatures and pressures have been established as the optimum conditions for alpha-amylase and glucoamylase activities in their respective medium. More so, alpha-amylase and glucoamylase-based hydrolysis are associated with a high rate of glucose yield, exceptional resistance of the enzymes to denaturation, especially at high temperatures, and reduced viscosity of the starchy medium^19^. *Saccharomyces cerevisiae* was selected as the fermentation organism for its cost-effectiveness and efficient glucose conversion^20^. The ideal fermentation conditions (temperature of 32 °C and pressure of 1.013 bar for 48 h) were selected^21^.

Double-effect distillation columns were the chosen distillation technology as they promote energy savings at a low cost^22^. while the first column performed at best condenser temperature (107.6 °C) and reboiler *condition* (111.3 °C pressure is 1.013 bar), the other performed at condenser temperature (84.1 °C) and reboiler condition (99.9 °C and pressure of 1.013 bar) for ethanol recovery. This approach concentrated the bottom of the first column and solids of the pretreatment section in a multi-effect evaporator. The dried solid was collected to be sold as animal feed. The bottom of the second column and the evaporator joined the distillation feed stream. The cost of the hydrophilic membrane, the complexity of installing pervaporation, and solvents recovery costs for azeotropic and extractive distillations^23^ favour the selection of molecular sieve adsorption process (operating at a contact time of 7.36 min, approach speed of 529.088 cm/s, the temperature of 85 °C and pressure of 1.013 bar) for the four processes in dehydration.

### Process simulation and economic analysis

Having selected the technologies as described in “Process technology selection” section a continuous process of 90% (329 days = 7896 h) annual running time^24^ was input in the Superpro Designer simulation startup window. The process flow models for the selected technologies were represented in the Superpro Designer simulation environment. Updated engineering plant and raw material costs were estimated using cost indices for November 2016^25^ which were 100.3 and 99.7 respectively, and November 2013 (106.8) as the base cost using Eq. (1).

1 $$\mathrm{Cost\,in\,year\,Y}=\mathrm{Cost\,in\,year\,X }\frac{\mathrm{Index\,in\,year\,Y}}{\mathrm{Index\,in\,year\,X}}$$

where, X = year 2013, Y = November, 2016.

The economic parameters extracted from Sinnott et al*.*, (2005) as given in Table 1 were input in the ‘Economic Evaluation Parameter’ window, and a startup period of 4 months was input into the ‘Time Valuation’ window^24^. The discounted cash flow parameters presented in Table 2 and the discount rate of 15.5%^26^ were input on the ‘Finances’ window. The indirect and fixed costs presented in Table 1 were input on the ‘Main Section’ window comprising capital cost and operating cost adjustments panes. The cost and size of equipment on flow models were registered on ‘Equipment Data’ panes.

**Table 1:** Assumed parameters’ values.

Parameter	Assumptions
**Indirect cost**	
Engineering and supervision	8% of TIE
Legal expenses	2% of TIE
Construction and contractor fee	15% of TIE
Project contingency	10% of TIE
Working capital	15% of TIE
Total capital investment (TCI)	TIE + indirect cost
**Fixed cost**	
Maintenance	7% of TCI
Operating labour	15% of the product cost
Laboratory cost	15% of operating labour
Operating supplies	15% of the maintenance cost
Supervision	10% of operating labour
Local taxes	2% of TCI
Insurance	1% of TCI
Plant overhead	60% of (operating labour + supervision + maintenance)

Source:^36^.

*TIE* total installed equipment.

**Table 2:** Parameters used for discounted cash flow calculations.

Parameter	Assumption
Discount rate	15.5%^a^
Plant lifetime	10 years
Working capital	15% of fixed capital investment
Construction period	3 years
Project life	10 years
Tax rate	30%^b^
Depreciation	Straight line method

Sources: ^a^^26^, ^b^^36^.

Similarly, the costs of feedstocks were registered on the ‘Pure Component’ pane. The prices were registered on a per-kilogram basis and the price of the products (ethanol) was set at $3.27 per gallon^27^. The average cost of co-product (electricity) was set at $0.047/kWh. Utility steam and cooling water requirement costs within the programme were set at US$ 17.08 per 1000 kg and US$ 0.1 per 1000 kg respectively^11^. The exchange rate used was $ = ₦437^26^. The costs of major equipment were obtained from equipment suppliers and erectors^28^. Equipment of varying capacities from received quotations had their costs adjusted using the scaling expression given by Eq. (2).

2 $$C=Co\mathrm{x}{\left(\frac{Q}{{Q}_{b}}\right)}^{n}\left(\frac{I}{{I}_{o}}\right)$$

where C = current cost, dollars, C_0_ = base cost, dollars, Q = current capacity, Q_b_ = base capacity, I = current index, dimensionless, I_o_ = base index, dimensionless n = exponent number or cost factor.

Prices of cassava, sweet potato, maize, and sorghum per kg are presented in Table 3. The equivalent revenue generated from the sales of the pomases and dried distiller grains with solubles (DDGS) resulting from their conversions was also considered. The carbon dioxide produced was released because of the high cost of purification and transportation to end users.

**Table 3:** Availability and cost of first-generation bioethanol feedstocks in Nigeria.

Feedstock	Quantity (tonnes/annum)	Market price ($) per kg	Bioethanol^a^ yield (litre/tonne)
Cassava	45,000,000^g^	0.05^g^	175^a^
Maize	8,180,000*	0.3*	360^a^
Sweet potato	2,700,000*	0.22*	125^a^
Sorghum	7,200,000^g^	0.19^h^	410^a^

Sources: ^a^^20^; ^g^^37,38^; ^h^^39^; *^40^.

### Environmental assessment

A life cycle approach of cradle-to-grave was chosen as the assessment approach using openLCA sustainability software. This method followed the product from its primal production stage of raw materials production through to its end use. The required information was the source of material and process parameters input (agrochemicals in Table 3, raw materials and utilities obtained from Superpro Designer, and distance traveled) and output (ethanol and electricity) within the openLCA system boundary. The system boundaries included:

i. Agricultural production of the feedstocks
ii. Feedstocks transportation to the processing site
iii. Feedstocks processing to fuel-grade bioethanol and coproducts (distillers dried grain with soluble and pomace).

Literature provided the types and amount of agrochemical input listed in Table 4. On activation, the openLCA window provided flow, process, and product interface. The flow interface was created and defined as ‘bioethanol flow’ for the four processing plants. This command led to process creation which was defined as a ‘bioethanol production process. The input and output, administrative information, parameters, allocations, and social aspects are found in the process creation window. A truck (> 35tonnes a trip from the openLCA database) with an assumed distance of 400 km considering traffic and the nature of Nigerian roads was selected. Materials for feedstock conversion were the input materials represented in openLCA while quantified bioethanol and electricity produced were registered as the output materials.

**Table 4:** Agrochemical inputs for grain and tuber starchy feedstocks.

Materials	Grain		Tuber	
	Corn	Sorghum*	Cassava^a^	Sweet potato^b^
Nitrogen N (kg/ha)	140	120	35.8	185
Phosphorus P (kg/ha)	100	113	33.4	46
Potassium K (kg/ha)	110	128	27.1	41
Liming material (kg/ha)	500	281	320	185
Herbicides (litres/ha)	13	3	**–**	–
Insecticide (litres/ha)	2.2	3	–	2
Formicide (litre/ha)	0.5	–	–	–

Sources: ^a^^16^, ^b^^41^, *^42^, – = not require.

After adding input and output materials, the ‘product system’ interface was created. A new window arose for the choice of editing the parameters, input, or output data before calculating the various associated impacts. Characterisation model (CMLbaseline 2016 v2.05) for life cycle impact assessment (LCIA) incorporated in SimaProv8.0 was imported into openLCA 1.5.5 and selected as the life cycle impact method. The CML baseline characterization model was then used to run the environmental assessment results where acidification, eutrophication, global warming, human toxicity, and photochemical oxidation potentials of the four bioethanol production processes were estimated.

The environmental and economic performances of the four production routes were then established from environmental emissions/discharge and energy perspectives (net energy ratio and energy renewability). The costs of all the environmental emissions/discharge for the four selected starchy feedstocks were then estimated and added to the annual operating cost of each process. Economic parameters such as gross margin, return on investment, net present value (NPV), internal rate of return (IRR), and payback period were re-estimated to establish the financial implications of the pollutants.

### Exergy/lost work analyses

Exergy analyses of the four production routes were conducted with Microsoft Excel spreadsheet using data from Superpro Designer simulations. Superpro Designer is weak with thermodynamic analysis, so Eq. (3)^9^ was used to estimate the total entropy of each unit. Evaluation of lost work and exergy components with Eqs. (3) to (5)^30^ followed subsequently. The exergy data were used to estimate the net energy ratio and renewability of the four-bioethanol production routes (see Eqs. 6 and 7).

4 $${\text{Lost}}\;{\text{ work}} = {\text{T}}_{{\text{o}}}\Delta {\text{S}}$$

5 $${\text{Total}}\;{\text{exergy}} = \Delta {\text{H }} - {\text{ T}}_{{\text{o}}} \Delta {\text{S}}$$

6 $${\text{Net}}\;{\text{energy}}\;{\text{ratio}}\;\left( {{\text{NER}}} \right) \, = \frac{Energy\;of\; the\; bioethanol\;produced}{{Energy\; consumed\; in\; the\; production\; of\; bioethanol}}$$

7 $${\text{Renewability}} = \frac{Energy\; of\; the\; bioethanol\; produced }{{Fossil\; fuel\; energy\; consumed}}$$

## Results and discussion

### Economic analysis

**Figure 1:**
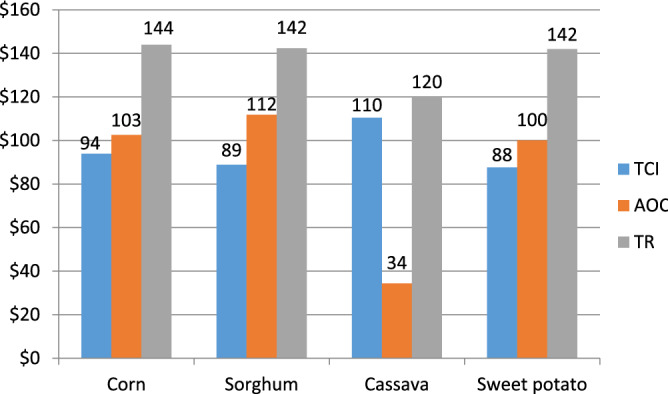
TCI, AOC, and TR of bioethanol produced from selected starchy feedstocks. *TCI* total capital investment, *AOC* annual operating cost, *TR* total revenue.

Figure 1 presents the total capital investment (TCI), annual operating cost (AOC), and total revenue (TR) of the processes that convert corn, sorghum, cassava, and sweet potato to fuel-grade bioethanol.

From Fig. 1, the cassava-based plant required the highest TCI of 110 million dollars while the TCI of the other three processes fell between 88 and 94 million dollars. This variation in TCI of the four processing plants was largely due to the higher raw materials requirement of the cassava process, which is a consequence of its relatively lower bioethanol yield of 175 kg ethanol/tonne (see Table 3). The economic impact of this difference in raw material requirement was less pronounced in the annual operating costs of the cassava-to-bioethanol process. This is because of the relatively low market price of cassava in Nigeria (Table 3).

**Figure 2:**
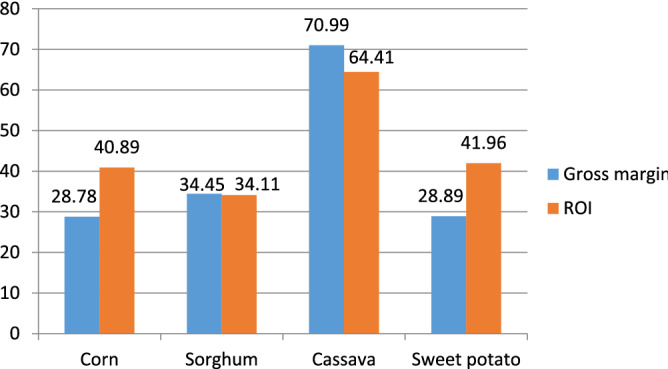
Gross margin and return on investment (ROI) of bioethanol produced selected starchy feedstocks.

Figure 2 shows the four-bioethanol production routes' gross margin and return on investment (ROI). ROI as a measure of profitability is the fraction of a process's profit after tax (PAT) and total capital investment (TCI). Cassava-based bioethanol plant gave the highest return on investment (ROI) value of 64.41% followed by sweet potato which recorded an ROI value of 41.96%. This ROI value showed that cassava is a more economically viable feedstock for bioethanol production among the four starchy feedstocks under study followed by sweet potato.

This economic viability of cassava and sweet potato was justified by their discounted payback periods (1.5 and 2 years) and net present value (NPV) ($151,964,521 and $124,516,463) at an annual discounted rate of 15.5% (Central Bank of Nigeria report, 2022) for the four bioethanol production routes and a project lifespan of ten (10) years (see Table 5). The NPV is a measure of the current value of future cash flows. The four processes assessed recorded positive NPVs as generated with Superpro Designer software for the economic analyses. These results signify the degree of viability of the investments concerning future cash flow. The NPVs presented in Table are the overall financial involvement of the four bioethanol production pathways under reference over their lifetime with cognizance of the time value of money. The observed differences in the NPV resulted from the different initial capital investments due to the variation in equipment sizing of the four processes. The equipment sizing is a factor of raw materials requirements to produce the desired throughput (158,000 m^3^/annum bioethanol) from each of the feedstocks, which ultimately affects the utility cost, annual operating costs, and the overall profitability of the processes. Similarly, the internal rate of return for each of the four processes was higher than the interest rate (15.5%) from which the NPV was generated (see Table 5).

**Table 5:** Net present value, internal rate of return, and discounted payback period.

Feedstock	Net present value ($)	Internal rate of return (%)	Payback period
Corn	86,789,943	19	2.5
Sorghum	72,627,563	17	3
Cassava	151,964,521	24	1.5
Sweet potato	124,516,463	34	2

The sensitivity analyses were conducted on the four fuel-grade bioethanol production pathways with Microsoft Excel and the results showed that the desired net profit can be realized only when the plants are operated at a minimum of 80% capacity utilization. Although the profit can still be realized through a price increase of the fuel produced. However, in this case, affordability is of the essence for the gasohol policy and its implementation to rapidly gain general acceptance for environmental sustainability. The sensitivity analysis also gave a break-even point at a bioethanol price of $3.27 per gallon, beyond which positive net present values were obtained for the four processes. It can therefore be asserted based on NPV, discounted payback period, and IRR that these four processes are economically profitable and acceptable.

### Environmental assessment

The environmental impact assessment was conducted using a life cycle approach of cradle-to-grave with openLCA sustainability software. This method followed the product from its primal production stage of raw materials production through to its end use. The openLCA software gave reports of global warming potential, acidification potential, eutrophication potential, photochemical oxidation potential, and human toxicity potential at a standard error of 0.077.

#### Global warming potential (GWP100)

**Figure 3:**
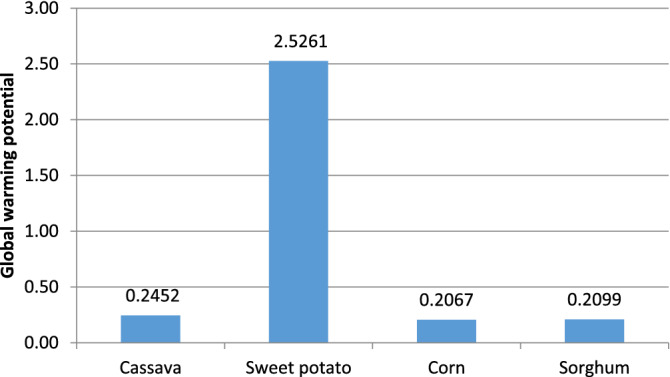
Global warming potential.

From Fig. 3, the net overall greenhouse gas emissions per kg of ethanol produced (measured relative to kg CO_2_ equivalents) are 0.2453, 2.5261, 0.2067, and 0.2099 for cassava, sweet potato, corn, and sorghum respectively. The results show that the global warming potential was highest in sweet potatoes with carbon dioxide from fossils contributing the highest emission of 85.19% (see Table 6). This is due to the transportation of large quantities of sweet potatoes required for the process, tractor operations, and nitrogenous fertilizer application.

**Table 6:** Percentage contribution of pollutants to environmental impact.

Feedstocks used for bioethanol production				
	Cassava	Sweet potato	Corn	Sorghum
**(GWP) Emission contributions (%)**				
Carbondioxide, fossil	66.93	85.19	50.89	46.70
Methane, fossil	0.01	0.05	0.05	0.09
Nitrogen monoxides	32.8	13.87	48.15	51.16
Carbon monoxide	0.16	0.89	0.91	1.87
**(AP) Emission contributions (%)**				
Sulfuric acid	87.71	48.27	0.0	0.0
Ammonia	7.44	26.85	55.89	51.90
Nitrogen dioxide	0.01	0.05	0.05	0.09
Nitrogen monoxides	4.68	23.87	43.15	46.14
Sulfur dioxide	0.16	0.96	0.91	1.87
**(EP) Emission contributions (%)**				
Ammonia	38.75	4.48	31.14	34.59
Dinitrogen monoxide	32.24	3.73	34.23	28.78
Nitrogen dioxide	0.05	0.01	0.03	0.08
Nitrogen oxide	28.96	4.74	34.60	36.56
Phosphorus	0.0	87.04	0.0	0.0
**(POP) Emission contribution (%)**				
Octane	100.00	0.0	100.00	99.75
Ammonia	0.0	99.95	0.0	0.02
Sulfur dioxide	0.0	0.05	0.0	0.23
**(HT) Emission contribution (%)**				
Ammonia	3.97	2.84	4.91	3.74
Nitrogen dioxide	0.16	0.19	0.13	0.09
Nitrogen oxide	95.77	96.84	94.87	95.14
Sulfur dioxide	0.10	0.13	0.09	1.03

The application of chemical fertilizers and herbicides generally improves crop production. However, concerns have been raised not only about the severe environmental problems posed by such practices but also about their long-term sustainability^29^. On the other hand, the use of organic materials (e.g., animal manures, crop residues, green manures, etc.) as an alternative source holds promise. Organic farming has been expanding at an annual rate of 20% in the last decade^31^.

#### Acidification potential (AP)

**Figure 4:**
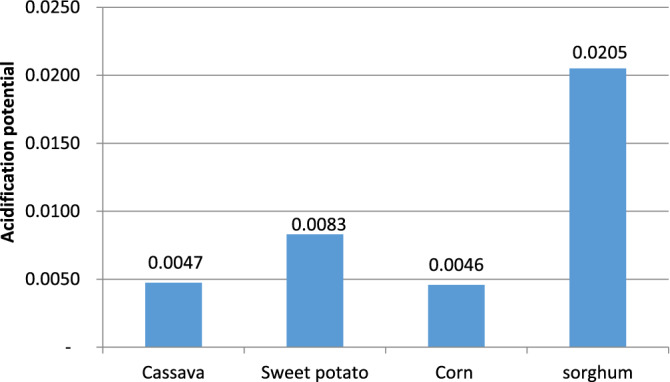
Acidification potential.

Enzyme production had recently been reported to be the dominant contributor to the overall acidification burdens due to SO_2_ emissions from fossil fuel consumption in bioethanol production processes^32^. In this work, the results presented in Fig. 4 show that the process utilising sorghum recorded the highest acidification potential value of 0.0205 kg SO_2_ equivalent.

A large amount of nitrogenous fertilizer required to produce the needed sorghum for the set bioethanol throughput (see Table 4) is responsible for the high acidification value recorded in the sorghum process. Ammonia and nitrogen monoxide were the dominant emission contributors of 51.90% and 46.14% respectively as shown in Table 6. The sulfuric acid used in the hydrolysis of the starchy feedstocks also contributed to the acidification burden on the environment.

#### Eutrophication potential (EP)

Eutrophication potential is generally associated with the environmental impacts of excessively high nutrients (such as N and P) that lead to shifts in species composition and increased biological productivity^33^.

**Figure 5:**
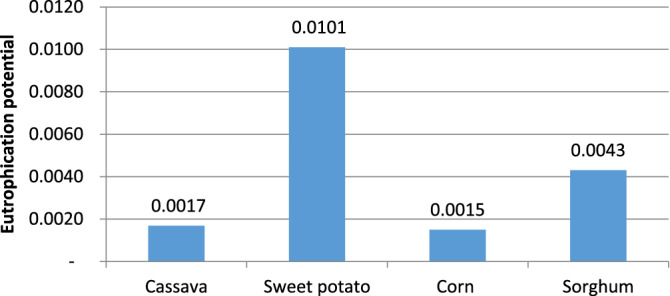
Eutrophication potential.

Among the bioethanol production processes assessed, bioethanol produced from sweet potatoes was the dominant contributor to the eutrophication burden as seen in Fig. 5. From Table 6, phosphorus constituted 87.04% of the overall emissions in the sweet potato process due to excessive liming material application, which is consequent to its relatively low yield of bioethanol.

Although corn, sorghum, and cassava require larger amounts of liming material, the overall quantity of sweet potato needed to actualize the desired bioethanol throughput is larger because of its lower bioethanol yield (Table 3).

#### Photochemical oxidation potential (POP)

**Figure 6:**
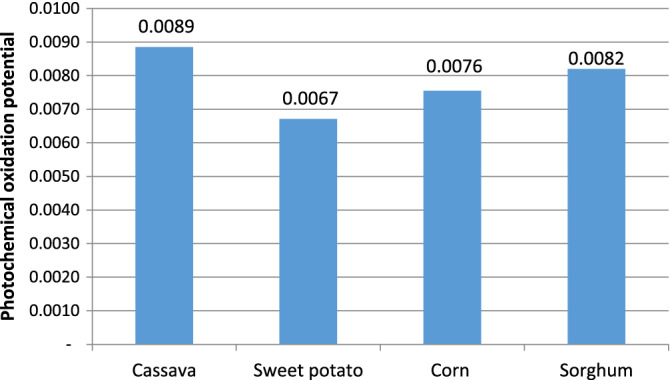
Photochemical oxidation.

Photochemical oxidation also referred to as summer smog is the result of reactions between NO_x_ and hydrocarbons or volatile organic compounds (VOCs)^33^. The four selected processes showed close photochemical oxidation burdens of 0.0089, 0.0067, 0.0076, and 0.0082 kg ethylene equivalent for cassava, sweet potato, corn, and sorghum respectively (Fig. 6). However, the photochemical oxidation impact of the cassava process was slightly higher due to excessive nitrogenous fertilizer utilization and excessive raw material requirements for the set throughput. Octane emission was the only contributor to the cassava and corn processes (Table 6).

#### Human toxicity potential-(HTP)

**Figure 7:**
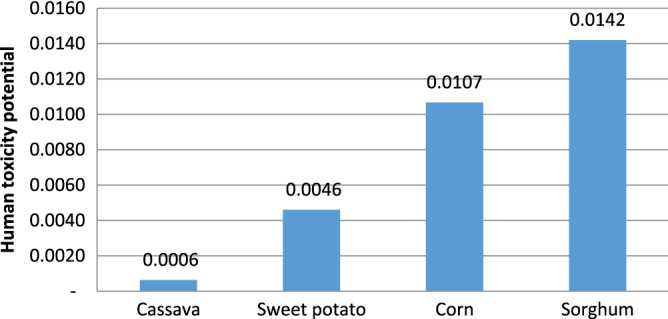
Human toxicity potential.

The human toxicity potential (HTP) of each emission of the emitted toxic substance into the air, water body, and soil is often measured relative to 1,4-dichlorobenzene (1,4-DB) and is expressed as kg 1,4 DB equivalent. The principal HTP in the four processes was derived from the hydrolysis phase, which involves the use of acid. Sorghum was the dominant contributor to human toxicity burden (recording 0.0142 kg 1,4-DB equivalent as shown in Fig. 7) with nitrogen oxide contributing about 95.14% as shown in Table 6, while cassava, corn, and sweet potato recorded 0.0006, 0.0046, and 0.0107 kg 1,4-DB equivalent respectively.

**Figure 8:**
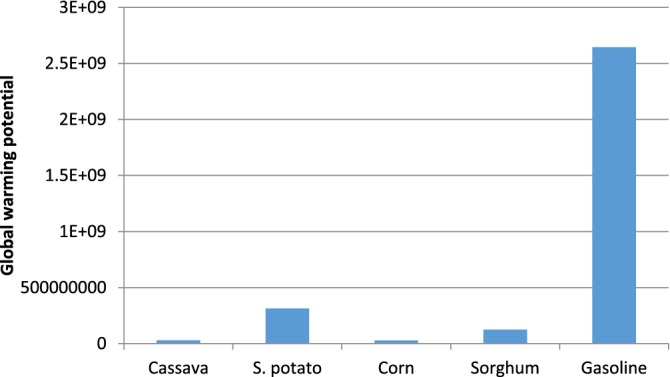
Comparison of the global warming potentials of the four routes with gasoline.

Figure 8 compares the global warming potentials of the four-bioethanol production routes with that of gasoline production processes. The differences between all four routes and gasoline production are quite pronounced.

**Figure 9:**
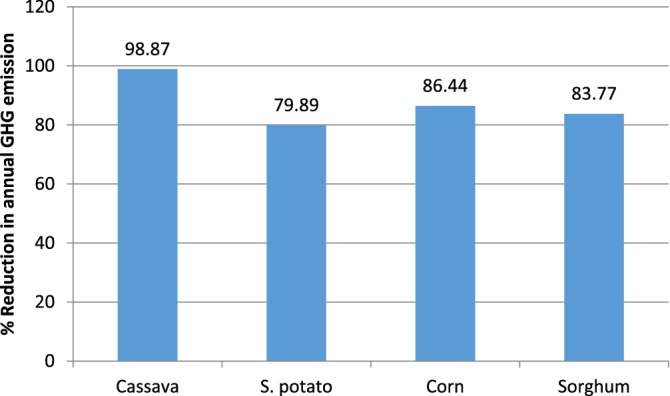
Reduced GHG emission per annum for the four production routes.

The sweet potato process recorded the highest value of greenhouse gas (GHG) emissions among the four processes. This can be attributed to fossil fuel combustion derived from tractor activities (such as bush clearing, ploughing, planting, mineral fertilizer application, and harvesting). The impact of the high global warming burden of the sweet potato process was also demonstrated in Fig. 9 where the “avoided emissions” is least for the bioethanol production process utilising sweet potato.

The environmental performances (concerning global warming, acidification, eutrophication, photochemical oxidation, and human toxicity burdens) of bioethanol production processes from the selected feedstocks were critically assessed and the processes were ranked accordingly as shown in Table 7.

**Table 7:** Ranking of the environmental impact of the selected bioethanol feedstocks.

Rank	Global warming	Acidification	Eutrophication	Photochemical oxidation	Human toxicity	Overall performance
1	Corn	Corn	Corn	Sweet potato	Cassava	Corn
2	Sorghum	Cassava	Cassava	Corn	Sweet potato	Cassava
3	Cassava	Sweet potato	Sorghum	Sorghum	Corn	Sweet potato
4	Sweet potato	Sorghum	Sweet potato	Cassava	Sorghum	Sorghum

The high level of consistency displayed by corn and cassava through economic and environmental sustainability assessment indicators proves that they are the most viable of the selected four starchy feedstocks. Cassava and sweet potato stood first and second in the economic assessment with return on investment of 64.41% and 41.96%. However, the difference in the economic performances of corn and sweet potato is insignificant as evident from Fig. 2. This profitability of cassava and corn processes matched their environmental performances, recording the least environmental burdens from their respective categories (see Figs. 4, 5, 6, 7 and 8) and the best avoided greenhouse gas emission result (see Fig. 9). It is obvious from the results that corn showed attractive economic trends but was partly overshadowed by its net energy balance (NEB) presented in “Net energy balance (NEB) and energy renewable” section.

### Net energy balance (NEB) and energy renewable

Table 8 presents the net energy balance (NEB) which is the ratio of the total energy of the bioethanol produced to the net energy consumed in the course of bioethanol production. From the results presented in Table 8, it can be seen that the bioethanol production process utilizing cassava was the most energy effective of the four processes.

**Table 8:** Net energy balance (NEB) of the bioethanol feedstocks.

Feedstocks	Net energy input (kWh)	Net energy output (kWh)	NEB
Cassava	104,447,746	779,456,459	7.5
Sweet potato	215,167,669	779,456,459	3.6
Corn	417,890,437	779,456,459	1.8
Sorghum	340,410,803	779,456,459	2.3

Table 9 depicts the energy renewability of the selected feedstocks. This is a ratio of the energy of the fuel-grade bioethanol produced to the net fossil energy consumed. The results showed that sweet potato and cassava consumed the least amount of fossil energy among the four production pathways. This observation could be traceable to the fact that sweet potato does not require the addition of herbicides and formicide (see Table 4).

**Table 9:** Energy renewability.

Feedstocks	Net fossil energy input (kWh)	Net energy output (kWh)	Renewability
Cassava	29,351,309	779,456,459	26.6
Sweet potato	9,873,992	779,456,459	78.9
Corn	23,892,505	369,995,154	15.5
Sorghum	37,968,989	369,995,154	9.7

Table 10 presents the pollutant emission and discharge costs used in this work.

**Table 10:** Pollutants and disposal costs.

S/N	Pollutants	Cost ($/tonne)
1	CO_2_	15
2	SO_2_	1.2
3	Phosphate	41
4	1,4-DB	51.8
5	Ethylene	7.4

Sources: Alliance for Jobs and Clean Energy^43,44^.

From Table 11, carbondioxide constitutes over 90% of the total emission costs for all the bioethanol production processes studied in this work. Sweet potato and cassava processes recorded the highest emissions of carbon dioxide of 4 691 641 and 454 766 tonnes/annum respectively. From the material balance results, the cassava conversion process to fuel-grade bioethanol produced 1625 tonnes/annum CO_2_ out of the 30 317 tonnes/annum, while that of the sweet potato process produced 1374 tonnes/annum CO_2_ out of 312 776 tonnes/annum as shown in Table 11. The remaining 28 692 and 311 402 tonnes/annum of CO_2_ from cassava and sweet potato processes emanated from fossil fuel combustion derived from bush clearing, ploughing, planting, mineral fertilizer application, harvesting, and subsequent transportation of the feedstocks to the processing site as summarized in Table 11. This fossil fuel-derived CO_2_ emission constituted a system bottleneck in the feedstock production phase.

**Table 11:** Pollutants and cost of emissions.

Cassava				Sweet potato		
Pollutants	Amount (tonne/annum)	Cost ($)	%Cost	Amount (tonne/annum)	Cost ($)	%Cost
Sulfur dioxide	585	703	0.15	974	1169	0.02
Carbon dioxide	30,317	454,766	95.49	312,776	4,691,641	98.12
Phosphates	212	8689	1.82	1276	52,351	1.09
1,4-Dichlorobenzene	75	3874	0.81	576	29,861	0.62
Ethylene	1109	8210	1.72	880	6514	0.14
	Total	476,243		Total	4,781,537	

Corn				Sorghum		
Sulfur dioxide	648	778	0.19	572	686	0.16
Carbon dioxide	25,772	386,577	96.66	26,166	392,498	90.83
Phosphates	37	1533	0.38	24	982	0.23
1,4-Dichlorobenzene	67	3462	0.87	598	30,996	7.17
Ethylene	1022	7564	1.89	941	6965	1.61
	Total	399,915		Total	432,126	

Upon assigning emission/discharge costs to the environmental pollutants as presented in Table 11, the overall emission cost of each processing plant was added to its annual operating cost and the economic parameters were re-evaluated as presented in Table 12. It was found that cassava maintained its status as the most economically viable bioethanol feedstock among the starchy feedstocks under study. Although there was a slight decrease in its ROI values (see Table 12). The economic profitability of the sweet potato process was greatly reduced by excessive carbondioxide emission and therefore, outperformed by the corn-to-bioethanol process.

**Table 12:** Economic parameters re-evaluation.

Economic parameters	Cassava	Sweet potato	Corn	Sorghum
Total capital investment($)	110,470,384	87,608,538	93,899,826	88,829,236
Emission costs ($)	476,243.774	4,781,537	399,915	432,126
Initial operating cost ($)	34,329,598	99,977,336	102,559,617	111,766,362
Final operating cost ($)	34,805,842	104,758,874	102,959,532	112,198,488
Final gross margin (%)	70.08	24.8	28.50	21.19
Initial return on investment (%)	64.41	41.96	40.89	34.11
Final return on investment (%)	54.11	36.51	40.59	33.77
NPV($)	151,964,521	124,516,463	86,789,943	72,627,563
IRR (%)	34	24	19	17
Initial payback time	1.55	2.38	2.5	3
Final payback period	1.9 ~ 2	2.7	2.49 ~ 2.5	2.96 ~ 3

### Exergy analyses

**Figure 10:**
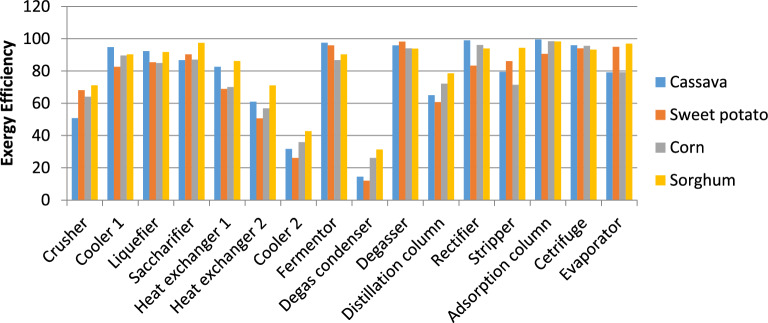
Exergy efficiency for starchy feedstocks conversion process.

Figure 10 depicts the exergy efficiencies of the four bioethanol production routes using the values generated with Superpro Designer in a Microsoft Excel spreadsheet. The highest exergy efficiencies for cassava, corn, and sorghum processes were observed in the adsorption columns. This is understandable as the interaction of the fluid in the adsorption column is largely mass transfer with little or no heat exchange. This varied slightly for a sweet potato-to-ethanol process where the highest exergy efficiency was observed at the degasser section due to the negligible difference in temperatures of utility high-pressure steam and the preheated fermentation broth. Degas vent condenser units recorded the least exergy efficiencies for all four-bioethanol production plants. This low efficiency can be attributed to the relatively high heat energy content of the carbondioxide leaving the degas condenser which causes most of the heat supplied by steam to be lost.

### Lost work analysis

Table 13 presents the lost work results obtained from processing the four-bioethanol feedstocks. From the results obtained, distillation columns constituted the highest lost work of over 50% in all four processes (see Table 13). The sorghum plant recorded the highest total lost work among the four starchy feedstocks. This is traceable to higher materials and utility requirements in the sorghum process. The corn processing plant recorded the least total lost work of 64 MW and therefore outperformed cassava, sweet potato, and sorghum from an energy efficiency perspective. This high-energy loss in the distillation column was therefore identified as the system bottleneck in the feedstocks processing phase for the four-bioethanol production processes considered in this work. Hence, it can be inferred that cassava and corn are the best bioethanol feedstocks upon a considerable tradeoff among economic, energy, and environmental perspectives.

**Table 13:** Lost work in starchy feedstocks conversion to bioethanol.

Units	Name	Cassava		Sweet potato		Corn		Sorghum	
		Lost work (kW)	% Lost work	Lost work (kW)	% Lost work	Lost work (kW)	% Lost work	Lost work (kW)	% Lost work
307 V	Crusher	7372.7947	7.23	7810.82	6.89	3686.4	5.75	3351.27	4.74
HX101	Cooler 1	9841.6289	9.65	8765.09	7.74	7029.73	10.96	6930.72	9.80
310 V	Liquefier	62.4025	0.06	115.71	0.10	39.0016	0.06	52.0021	0.07
321 V	Saccharifier	620.0344	0.61	760.00	0.67	326.334	0.51	295.254	0.42
402E	Cooler 2	3797.7954	3.72	4014.12	3.54	3452.54	5.38	1808.47	2.56
405 V	Fermentor	1043.8086	1.02	1928.42	1.70	957.623	1.49	499.43	0.71
408E	Degas condenser	36.5682	0.04	39.02	0.03	30.4735	0.05	29.2546	0.04
412 V	Degasser	528.2596	0.52	719.25	0.64	484.642	0.76	409.504	0.58
501 T	Distillation column	61,009.9969	59.81	68,420.63	60.42	34,275.3	53.44	49,601.6	70.14
603 V	Adsorption column	4217.7141	4.14	6678.77	5.90	2636.07	4.11	1622.2	2.29
610D	Evaporator	13,467.8181	13.20	13,989.87	12.35	11,223.2	17.50	6121.74	8.66
Total		101,998.821		113,241.7048		64,141.28		70,721.48	

### Feedstocks production

Carbondioxide emissions in the feedstock production phase were derived from fossil fuel combustion from tractor operations (such as bush clearing, ploughing, and planting, mineral fertilizer application, pesticide application, harvesting, and processing) as estimated in Table 14. Mineral fertilizer usage is the major contributor to CO_2_ emission in corn production with about 62% of the overall CO_2_ released^31^ while a similar process in cassava production contributes about 30.15% of CO_2_^34^. Hence, mineral fertilizer application was identified as a feedstock production phase bottleneck. According to the findings of Tongwane et al*.* (2016), and Nguyen et al*.* (2007), the use of compost manure derived from decayed plant residue left in the field is capable of reducing CO_2_ emission by 62% and 30.15% for corn and cassava productions respectively with the corresponding reduction in annual operating costs by $129 760, and $72 475 respectively as expressed in Table 14. This is justifiable, as the crop yield with compost manure usage for various crops is higher compared to mineral fertilizers utilization^29^.

**Table 14:** Feedstock production phase debottlenecking.

Process plant	Feedstock production (tonne/annum)	Reduced emission (%)	Avoided CO_2_ (tonne/annum)	Reduced cost ($) ($15/tonne)
Cassava	28,692.198	30.15	8650.70	129,760.5
Corn	7793.116	62	4831.73	72,475.98

### Feedstocks processing

The feedstock processing phase bottleneck was identified to be the distillation column with 61 MW and 32 MW of lost work in cassava and corn processing plants respectively. These figures amount to 59.81 and 53.44% of the total energy losses in their respective processes. As a way of reducing energy losses in fermentation broth purification, Suleiman et al. (2014) suggested the use of a hybrid configuration of a totally heat-integrated distillation column (THIDC) and molecular sieve adsorption column^35^. His study on the exergo recycled stream-economic assessment of bioethanol refining showed that energy savings of 61.8% could be achieved with the use of THIDC and molecular sieve hybrid configuration. The adoption of this hybrid configuration in this work is capable of reducing the cost of utility by $2827.81 and $1588.66 for the cassava and corn processing plants respectively as presented in Table 15.

**Table 15:** Feedstock processing phase debottlenecking.

Process plant	Feedstock processing lost work (kW)	Energy savings (%)	Avoided energy lost (kW)	Reduced cost ($) ($0.075/kW)
Cassava	61,010.00	61.8	37,704.18	2827.81
Corn	34,275.3	61.8	21,182.14	1588.66

## Conclusion

A sustainability study of four selected starchy feedstocks (cassava, sweet potato, corn, and sorghum) for bioethanol production was conducted to include economic, environmental, and energy indicators. The four-bioethanol production routes were found to be economically viable recording ROI values of 64.41, 41.96, 40.89, and 34.11% for cassava, sweet potato, corn, and sorghum processes respectively. While all the processes recorded positive NPVs for a discounted rate of 15.5% and a 10-year project span, the sensitivity analyses revealed an important constraint to the four processes, which limit the capacity utilization of the production routes to a minimum of 80% per annum below which the projects recorded losses. The four processes were also observed to be environmentally favorable from an energy perspective as they recorded net energy balance values of greater than 1. The four processes portrayed appreciable economic viability (recording new ROI values of 54.11, 36.51, 40.59, and 34.11% for cassava, sweet potato, corn, and sorghum processes respectively) despite adjusting the economic parameters to include pollutants emission/discharge costs. Because of the huge impact, carbon dioxide emission cost had on the sweet potato process, the final ROI value of the sweet potato process fell below that of the corn-based bioethanol process. As such cassava and corn-based bioethanol production processes represent the two most sustainable bioethanol production routes. The two production routes (cassava and corn-based processes) recorded a total lost work of 101 MW and 64 MW respectively. As a way of enhancing the energy and environmental performances of the best two processes, which would ultimately translate into better economic prosperity, compost manure utilisation and hybrid configuring of thermally integrated distillation column (THIDC) were suggested to minimize CO_2_ emissions and lost work in the feedstock production and processing phases respectively. The major limitations to this research work are the insufficiency of a reliable database on costs of feedstocks and materials with their variations, fluctuations in currency exchange rates, and the cost of acquiring licensed software that contains all the information of the selected feedstocks. Further comparative sustainability studies concerning these three indicators (economic, environmental, and energy) should be conducted simultaneously on more attractive third and fourth-generation bioethanol feedstocks.

## Data Availability

The supporting data from which the results of this work were generated are openly available in the following links presented in Table

**Table 16:** Data source.

Data description	Source
Hydrolysis reaction conditions	http://www.afdc.doe.gov/pdfs/3955.pdf
Fermentation conditions	https://www.ijera.com/papers/Vol2_issue4/GI2411421151.pdf
Double-effect distillation column operating parameters	http://doi.org/10.1016/J.ENERGY.2010.09.024
Costs of pollutants emission	https://www.oecd.org/env/outreach/38118149.pdf
Availability and cost of bioethanol feedstocks	https://www.fao.org/unfao/procurement/statistics-from-2010-2020/statistics-2013/en/ www.fews.net/nigeria/special-report/december-13-2016 https://www.dailytrust.com.ng/harvest-prices-of-foodstuffs-in-kano.html
Agrochemical input data	https://www.nass.usda.gov/Surveys/Guide_to_NASS_Surveys/Chemical_Use/2013_Peanuts_Highlights/ https://www.researchgate.net/publication/43529567_Response_of_Sorghum_to_Nitrogen_Fertilizer_and_Plant_Density_in_the_Guinea_Savanna_Zone https://www.academia.edu/60415229/SEED_INOCULATION_WITH_Azospirillum_brasilense_ASSOCIATED_WITH_THE_USE_OF_BIOREGULATORS_IN_MAIZE
Molecular sieve adsorption parameters	https://www.nrel.gov/docs/fy02osti/32438.pdf
Economic parameters	https://books.google.com/books/about/Plant_Design_and_Economics_for_Chemical.html?id=3uVFkBBHyP8C Data source. https://www.fao.org/unfao/procurement/statistics-from-2010-2020/statistics-2013/en/ www.fews.net/nigeria/special-report/december-13-2016 https://www.dailytrust.com.ng/harvest-prices-of-foodstuffs-in-kano.html https://www.nass.usda.gov/Surveys/Guide_to_NASS_Surveys/Chemical_Use/2013_Peanuts_Highlights/ https://www.researchgate.net/publication/43529567_Response_of_Sorghum_to_Nitrogen_Fertilizer_and_Plant_Density_in_the_Guinea_Savanna_Zone https://www.academia.edu/60415229/SEED_INOCULATION_WITH_Azospirillum_brasilense_ASSOCIATED_WITH_THE_USE_OF_BIOREGULATORS_IN_MAIZE

## Abbreviations

FAO: Food and Agricultural Organisation
FEWS: Famine Early Warning System
